# siRNA-Like Double-Stranded RNAs Are Specifically Protected Against Degradation in Human Cell Extract

**DOI:** 10.1371/journal.pone.0020359

**Published:** 2011-05-27

**Authors:** John A. H. Hoerter, Vishalakshi Krishnan, Troy A. Lionberger, Nils G. Walter

**Affiliations:** 1 Department of Chemistry, University of Michigan, Ann Arbor, Michigan, United States of America,; 2 Department of Mechanical Engineering, University of Michigan, Ann Arbor, Michigan, United States of America; French National Center for Scientific Research - Institut de biologie moléculaire et cellulaire, France

## Abstract

RNA interference (RNAi) is a set of intracellular pathways in eukaryotes that controls both exogenous and endogenous gene expression. The power of RNAi to knock down (silence) any gene of interest by the introduction of synthetic small-interfering (si)RNAs has afforded powerful insight into biological function through reverse genetic approaches and has borne a new field of gene therapeutics. A number of questions are outstanding concerning the potency of siRNAs, necessitating an understanding of how short double-stranded RNAs are processed by the cell. Recent work suggests unmodified siRNAs are protected in the intracellular environment, although the mechanism of protection still remains unclear. We have developed a set of doubly-fluorophore labeled RNAs (more precisely, RNA/DNA chimeras) to probe in real-time the stability of siRNAs and related molecules by fluorescence resonance energy transfer (FRET). We find that these RNA probes are substrates for relevant cellular degradative processes, including the RNase H1 mediated degradation of an DNA/RNA hybrid and Dicer-mediated cleavage of a 24-nucleotide (per strand) double-stranded RNA. In addition, we find that 21- and 24-nucleotide double-stranded RNAs are relatively protected in human cytosolic cell extract, but less so in blood serum, whereas an 18-nucleotide double-stranded RNA is less protected in both fluids. These results suggest that RNAi effector RNAs are specifically protected in the cellular environment and may provide an explanation for recent results showing that unmodified siRNAs in cells persist intact for extended periods of time.

## Introduction

RNA interference (RNAi), first described in the nematode *C. elegans* [1], is a set of conserved post-transcriptional gene silencing pathways in eukaryotes. Following the first demonstration that RNAi is functional in human cells and receptive to using synthetic small interfering (si)RNA effector molecules [2], significant progress has been made in harnessing the RNAi pathway for functional genomics and for therapies targeting previously “undruggable” genetic targets [3]–[6]. Inside the cell, double-stranded siRNAs specifically interact with a number of proteins including the endoribonuclease Dicer that cleaves them from long precursor RNAs and steers incorporation of this product into a multiprotein complex referred to as the RNA-induced silencing complex (RISC). One strand of the siRNA duplex is retained in RISC and acts as a template for the sequence-specific identification and site-specific cleavage of messenger (m)RNAs and concomitant reduction in gene expression.

The inherent sensitivity of RNA molecules both inside and outside the cell towards degradation by ribonucleases motivated an immediate focus on development of chemically modified siRNAs that can withstand nucleolytic extracellular environments and reach the cellular milieu intact [3]. Investigations into the determinants of siRNA stability have focused on extracellular stability, intracellular stability, and potency of silencing. First, to mimic the extracellular environment several groups have used blood serum and purified nucleases to determine the sensitivity of siRNAs to degradation. Chemical modifications were found to impart a considerable degree of nuclease resistance to siRNAs while retaining the ability to induce silencing through the RNAi pathway [7]–[18]. Furthermore, chemical modifications were found to be absolutely essential for systemic delivery of siRNA, both to mediate binding to serum proteins to increase circulating half-life and to block sites vulnerable to nuclease attack [14,15]. Only few exceptions to this tendency towards RNA-hostile extracellular environments exist [19].

The intracellular milieu, which is the functional environment of the siRNA, is the second environment to which the siRNA is exposed. By monitoring the expression of a target gene, several groups were able to assess the potency and duration of siRNA effects, including a number of studies that focused on determining whether chemically modified siRNAs are more potent than unmodified siRNAs [7–11,13,16]. A recent study has shown, however, that enhanced intracellular nucleolytic stability is not necessarily correlated with increased duration of the silencing effect [8]. In fact, the authors found that silencing in non-dividing cells persisted for up to one month from a single dose of an unmodified siRNA, suggesting that siRNAs may be quite stable inside the cell.

Several groups have addressed questions of intracellular siRNA stability and localization by introducing fluorophore modified siRNAs into live cells and using various microscopy techniques [20]. siRNAs are actively exported from the nucleus [21], except in cases where the RNA target is located in the nucleus [22]. Fluorescence fluctuation spectroscopy has been utilized in a separate study to assess the integrity of labeled intracellular RNAs, revealing that doubly-labeled RNA suitable for fluorescence resonance energy transfer (FRET) measurement between the fluorophores is relatively unstable in single-stranded form compared to the corresponding siRNA duplex [23]. Intracellular FRET imaging of dsRNAs has also been employed to show that intact siRNA duplexes accumulate in cellular foci identified as P-bodies [24,25]. We have used FRET labeled single-stranded RNAs to show that secondary structure in general attenuates degradation in human cell extracts [26].

The precise origin of the apparent intracellular protection of siRNAs is unclear. Long duration of silencing in non-dividing cells may be indicative of the stability of the single-stranded guide strand incorporated into RISC. Prior to RISC maturation, it can be assumed that some stability is derived from the decreased nuclease sensitivity of double-stranded RNA compared to single-stranded RNA [23,26]. Previous intracellular fluorescence studies [21–25] have focused, however, only on canonical 21–22 nucleotide (nt, per strand) siRNAs, while the observation that the RNAi pathway can utilize 21- to 27-nt double-stranded RNAs [27] suggests that intracellular double-stranded RNAs are discriminated based on length.

**Figure 1:**
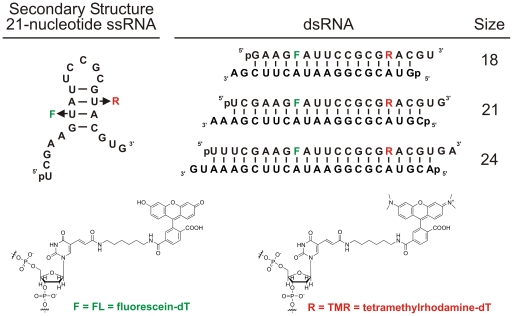
Design of the single- and double-stranded RNAs used in this study. The sequences for the RNAs are derived from the *pp-luc* firefly luciferase gene and the corresponding siRNA previously reported [12,28]. The double-stranded RNAs consist of an identical central sequence and internal FL and TMR modifications, and the flanking sequences are derived from the luciferase gene. All dsRNAs are fully Watson-Crick base paired and contain the 2-nt 3′ overhangs characteristic of siRNAs; “p” indicates 5′ phosphate.

To better evaluate the potential of siRNAs in gene therapeutics, a real-time characterization of their degradation kinetics under extracellular and intracellular conditions is necessary. Such an assay should include rapid and precise assessment of RNA stability as a function of bodily fluid, base paring partner, and RNA size and should eventually be amenable to high-throughput screening for optimizing siRNA drugs. To this end, we here have developed a generalizable series of RNAs modified for FRET measurements, where two uridines in positions 7 and 16 of the 21-nt guide strand were replaced with amino-modified deoxythymidines to introduce two fluorophores that undergo distance dependent FRET (**Figure** 1). We varied the size of the labeled strand to generate double-stranded (ds)RNAs ranging from 18–24 nt per strand (while keeping the fluorophore distance constant, **Figure** 1). We find that our dsRNA probes interact with enzymes in RNAi-active cytosolic extract from human HeLa cells, including RNase H1 (21-nt DNA/RNA hybrid) and Dicer (24-nt dsRNA). The DNA/RNA hybrid degradation by RNase H1 is quite efficient, with observed rate constants exceeding those for the single-stranded RNA, which is not expected to be an RNase H1 substrate. Furthermore, our experimental design allows us to correlate the previously studied potency/size relationships for siRNAs [27,28] with a real-time readout of RNA stability. Our results demonstrate that 21- and 24-nt dsRNAs are protected in cell extract relative to the 18-nt dsRNA, potentially contributing to the longevity of intracellular RNAi effects.

## Results

We here have developed a set of fluorescein (FL) and tetramethylrhodamine (TMR) modified RNAs (or, more precisely, RNA-DNA chimeras with two 2′-deoxy modifications necessary for fluorophore labeling; **Figure 1**) for use in studies of intracellular and extracellular RNA stability by real-time FRET. To specifically address the question of how double-stranded RNAs are recognized as substrates by intra- and extracellular ribonucleases, we developed three dsRNAs (**Figure 1**) of 18, 21, and 24 nt length that bracket the canonical human siRNA length and are derived from an siRNA previously developed and validated against the *pp-luc* luciferase gene [12,28]. We fluorophore labeled the strand that dominantly acts as guide for the RNAi machinery and is antisense to the luciferase mRNA [12,28]. We first sought to compare the degradation of these dsRNAs with those of the corresponding, relatively unstructured single-stranded RNA (ssRNA, **Figure 1**) and its hybrid with a fully complementary DNA under our standard conditions of near-physiologic buffer (50 mM Tris-HOAc, pH 7.4, 80 mM KCl, 20 mM NaCl, and 1 mM MgCl_2_) at 37°C.

### Double-stranded RNA is stabilized in both cell extract and serum

To establish the relative degradation of a 21-nt dsRNA compared to a DNA/RNA hybrid and ssRNA, we applied our steady-state FRET assay to compare the degradation of these complexes upon the addition of 30% (v/v) of either fetal bovine serum (FBS) or HeLa S100 cytosolic cell extract, equivalent to 11.1 mg/ml total serum and 2.49 mg/ml total cellular protein, respectively. The blood serum contains a significant fraction of RNase A-like enzymes [29], whereas the cell extract contains the protein components of the RNAi pathway and was previously tested as RNAi-active [12].

**Figure 2:**
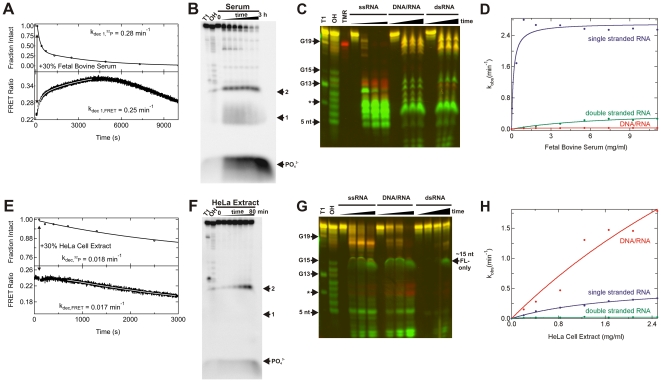
Monitoring the degradation of ssRNAs and dsRNAs in fetal bovine serum and HeLa S100 cytosolic cell extract. (**A**) Degradation time course of the 21-nt dsRNA in 30% (v/v) serum, as monitored either in real-time by steady-state FRET (bottom) or discontinuously by radioactive labeling and denaturing gel electrophoretic analysis of time points (top). Solid lines represent double-exponential fits to both types of degradation data. In both cases the faster of the two rate constants are reported here and elsewhere and demonstrate good agreement between the FRET and radioactive assays. (**B**) Denaturing radioactive gel electrophoretic analysis corresponding to the data in panel A (top). RNase T1 (“T1”) and alkali (“OH”) ladders were run alongside varying time points of RNA degradation to identify the products. Arrows denote bands corresponding to positions 1, 2 (from the 5′ end), and PO_4_^3−^. (**C**) Denaturing FRET gel revealing the serum degradation patterns of the indicated RNAs as a function of time, alongside RNase T1 (“T1”, leading to cleavage 3′ of G as indicated by arrows) and alkali (“OH”) ladders as well as a TMR-only labeled color calibration standard. The * denotes a double cut at both G13 and G3. (**D**) Observed degradation rate constants for the various RNAs as a function of total serum protein concentration, quantified from FRET time traces as exemplified in panel A (bottom). (**E**) Degradation time course of the 21-nt dsRNA in 30% (v/v) cell extract, as monitored either in real-time by steady-state FRET (bottom) or discontinuously by radioactive labeling and denaturing gel electrophoretic analysis of time points (top). Solid lines represent single-exponential fits to both types of degradation data with the respective rate constants shown, demonstrating good agreement between the FRET and radioactive assays. (**F**) Denaturing radioactive gel electrophoretic analysis corresponding to the data in panel E (top). RNase T1 (“T1”) and alkali (“OH”) ladders were run alongside varying time points of RNA degradation to identify the products. Arrows denote bands corresponding to positions 1, 2 (from the 5′ end), and PO_4_^3−^. (**G**) Denaturing FRET gel revealing the cell extract degradation patterns of the indicated RNAs as a function of time, alongside RNase T1 (“T1”, leading to cleavage 3′ of G as indicated by arrows) and alkali (“OH”) ladders. The * denotes a double cut at both G13 and G3. (**H**) Observed degradation rate constants for the various RNAs as a function of total cell extract protein concentration, quantified from FRET time traces as exemplified in panel E (bottom). Further details of the experiments are found in Materials and Methods.

In general, a decrease in FRET ratio should be indicative of degradation of the labeled strand, as cleavage between the fluorophore labels is expected to lead to a decrease in FRET efficiency. Surprisingly, when the dsRNAs were incubated in serum, we observed two fast phases representing increases in FRET ratio, followed by a slow decrease (**Figure 2A**). Assays using purified RNase A (the major type of ribonuclease present in serum) qualitatively reproduced this shape of the FRET ratio time trace (**Figure S1**). In cell extract, the fast phase(s) decreased significantly (**Figure 2E**). To assess which phase and corresponding rate constant from the FRET traces reflect RNA degradation, we used 5′-^32^P radiolabeling of the 21-nt fluorophore-modified guide strand and denaturing gel electrophoretic analysis [12] to determine the degradation rate constant independent of the real-time output of our FRET trace (**Figures 2B,F**). Gels were quantified for the fraction of the guide strand that was intact, therefore quantifying only primary cleavage events. The resulting data were plotted and fit with exponential functions (double-exponential increase for serum and single-exponential decrease for HeLa cell extract). Comparison with these data unambiguously identifies the relevant degradation rate constants from the FRET time traces as the dominant rise phases in serum and the dominant decrease phase in HeLa cell extract (**Figures 2A,E**). We note that the curvatures and rates of the radioactively monitored decay time courses are well reflected in the real-time FRET time traces (despite the fact that the FRET trace in serum is inverted), attesting to the validity of using FRET to monitor the integrity of the guide strand over time. The increase in FRET particularly upon degradation in serum may be due to, for example, transient formation of doubly-labeled, (partially) single-stranded RNAs with shorter fluorophore distances.

We next qualitatively showed by denaturing FRET gel analysis that serum manifests more efficient cleavage of the ssRNA compared to either the DNA/RNA or the dsRNA duplex (**Figure 2C**). While the cleavage patterns of the DNA/RNA hybrid and dsRNA are quite similar, the cleavage pattern of the ssRNA is notably different. These differences include accumulation of a slight TMR-only band in the ssRNA sample (note the similar color to the reference TMR-only control lane), which indicates selective removal of the FL donor fluorophore. There is also significant accumulation of intermediate size (5–13 nt) products that are either singly TMR or FL labeled. By contrast, products smaller than 5 nt are almost absent from the ssRNA lanes, suggesting primarily endonucleolytic, rather than 5′ exonucleolytic, decay (and/or very rapid degradation of smaller fragments). Some accumulation of short FL-only labeled products is seen upon degradation of the DNA/RNA hybrid and dsRNA, suggesting limited 5′ exonuclease activity.

The degradation patterns in HeLa cell extract are markedly different (**Figure 2G**). Most notably, the DNA/RNA hybrid is more completely degraded in HeLa cell extract, whereas the ssRNA is more completely degraded in serum (compare with **Figure 2C**). Another difference between serum and cell extract is the significant accumulation of short (<5 nt) products in the HeLa lanes, suggesting that these samples contain substantial detectable 5′ and 3′ exonucleolytic activities. The gel also shows that degradation of ssRNA by HeLa cell extract generates long (15–20 nt) exonucleolytic products that are not found for either the DNA/RNA hybrid or dsRNA (**Figure 2G**). There is also a FL-only labeled, somewhat diffuse band found in all samples incubated with cell extract that co-migrates with an ∼15-nt RNA fragment (**Figure 2G**). Given that the two fluorophores are attached only 8 nt apart (**Figure 1**) this band may be evidence of a tight interaction of a component of the cell extract with a shorter, FL-only labeled fragment, leading to a band shift in the denaturing gel.

Applying our real-time FRET degradation assay to the three fluorophore labeled RNA variants allowed us to determine their decay rate constants over a range of total protein concentrations from added serum or cell extract (**Figures 2** **D,H**). Consistent with our qualitative observations by gel electrophoresis, the fastest observed rate constants are found for the decay of ssRNA in serum (maximum velocity at saturation, v_max_ = 2.7 min^−1^; half-titration point FBS_1/2_ = 0.14 mg/ml; **Figure 2D**). Degradation rate constants are significantly slower upon formation of secondary structure, as the dsRNA (v_max_ = 0.47 min^−1^; FBS_1/2_ = 7.7 mg/ml) and particularly the DNA/RNA hybrid (v_max_ = 0.03 min^−1^; FBS_1/2_ = 0.46 mg/ml) are less efficiently degraded.

The relative decay kinetics in HeLa cell extract (**Figure 2H**) are significantly different from those in serum (compare with **Figure 2D**). Here the DNA/RNA hybrid is most efficiently degraded (v_max_ = 7.4 min^−1^; HeLa_1/2_ = 7.6 mg/ml; **Figure 2H**), while the ssRNA is relatively protected (v_max_ = 0.60 min^−1^; HeLa_1/2_ = 1.9 mg/ml). We find that the secondary structured dsRNA is most strongly protected in cell extract, leading to only a slow decay (v_max_ = 0.02 min^−1^; HeLa_1/2_ = 0.18 mg/ml). In both cell extract and blood serum, the formation of dsRNA thus protects ssRNA against nucleolytic degradation.

### DNA/RNA hybrid is degraded by RNase H1 in HeLa cell extract

**Figure 3:**
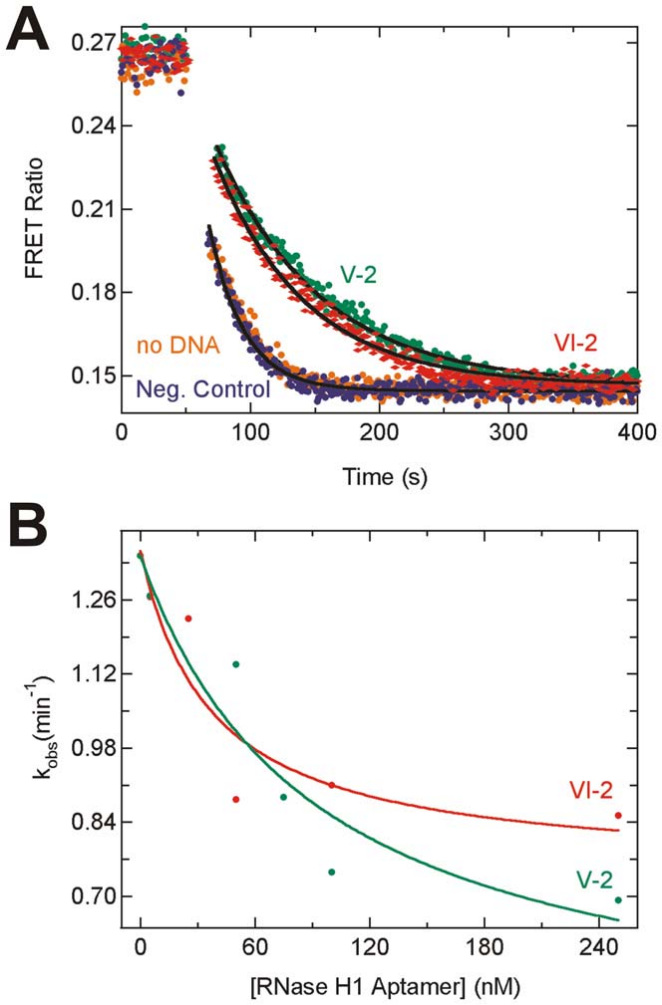
Inhibition of degradation of the doubly-labeled DNA/RNA hybrid in 30% (v/v) HeLa S100 cytosolic cell extract upon addition of DNA aptamers. (**A**) Steady-state FRET assays showing attenuation of degradation by RNase H1-specific DNA aptamers VI-2 (red) and V-2 (green) at 500 nM concentration. No such attenuation is observed in the absence of inhibitor (orange) or when 500 nM of the negative control S1-DNA is included (blue). Solid black lines represent single-exponential fits to the data to extract rate constants. (**B**) Rate constants of DNA/RNA degradation as a function of VI-2 or V-2 aptamer concentration. Solid lines are fits with a hyperbolic binding equation, revealing the nanomolar binding constants for the DNA aptamers reported in the text.

One of the most prominent features of our decay data is the fast degradation of the DNA/RNA hybrid in HeLa S100 cytosolic cell extract, contrasting with its strong protection in blood serum. We hypothesized that the efficient degradation of the DNA/RNA hybrid in cell extract results from the activity of human RNase H enzymes. RNase H activity degrades the RNA in a DNA/RNA hybrid and is important in nucleic acid processing in the cell, including in DNA replication. Two DNA aptamers have previously been *in vitro* selected for catalytic inhibition of human RNase H1 [30], allowing us to specifically test for RNase H1 degradation of the fluorophore-labeled DNA/RNA hybrid. **Figure 3A** shows the time evolution of the FRET ratio in several DNA/RNA hybrid degradation assays. In the absence of any added DNA or the presence of a negative control DNA, degradation is rapid. When the inhibitory RNase H1 aptamers VI-2 or V-2 were added, we observed a marked reduction in the observed decay rate constant, indicating specific inhibition of RNase H1 in the degradation of the fluorophore-labeled DNA/RNA hybrid. These experiments were repeated over a range of aptamer concentrations, and a dose dependent decrease in rate constant was observed for both aptamers (**Figure 3B**; VI-2: initial rate constant v_0_ = 1.35 min^−1^, minimal rate constant at saturation v_min_ = 0.75 min^−1^, half-titration point VI-2_1/2_ = 40 nM; V-2: v_0_ = 1.34 min^−1^, v_min_ = 0.4 min^−1^, V-2_1/2_ = 90 nM). These observations indicate that human RNAse H1 is (one of) the enzyme(s) responsible for degrading a DNA/RNA hybrid in HeLa cell extract.

### FRET labeled 24-nucleotide dsRNA is a Dicer substrate

**Figure 4:**
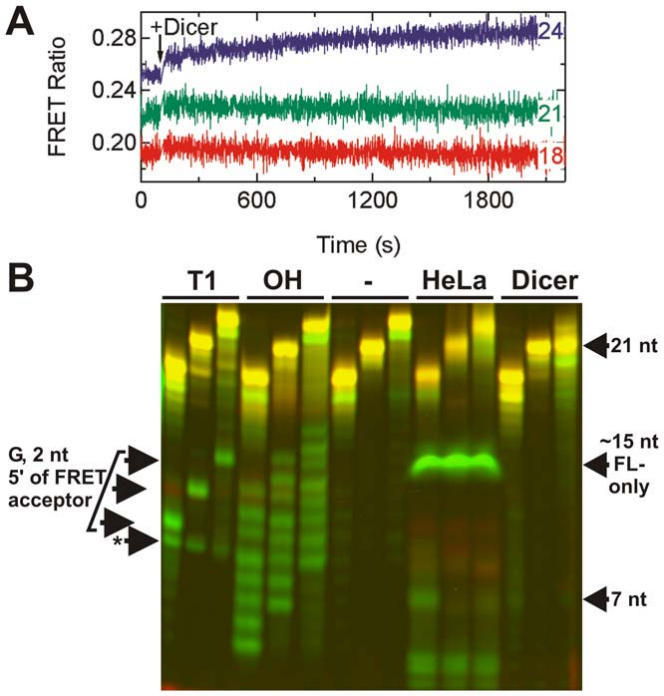
Processing of doubly-labeled dsRNAs of varying length by HeLa S100 cytosolic cell extract and purified human Dicer. (*A*) Steady-state FRET time traces of 18-, 21-, and 24-nt dsRNAs as indicated upon Dicer addition; only the 24-nt dsRNA time trace slightly increases. (*B*) Denaturing FRET gel revealing the degradation patterns of the 18-, 21-, and 24-nt dsRNAs (from left to right in each triplet of bands) as a function of time, alongside RNase T1 (“T1”, leading to cleavage 3′ of G as indicated by arrows on the left) and alkali (“OH”) ladders. “-” indicates incubation in buffer only, while the last two triplets of bands show samples incubated in HeLa cell extract and purified Dicer enzyme, respectively. Arrows to the right mark specific degradation products.

**Figure 5:**
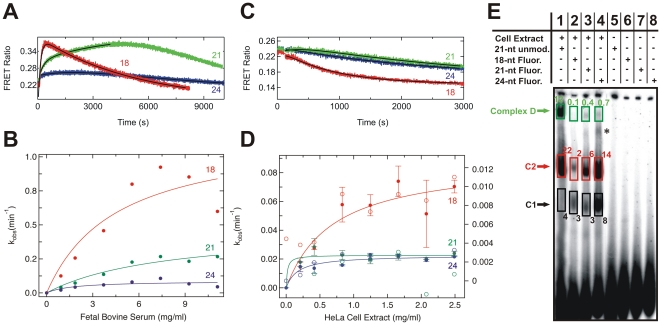
Degradation kinetics of 18-, 21-, and 24-nt dsRNAs in fetal bovine serum and HeLa S100 cytosolic cell extract. (*A*) Steady-state FRET time traces upon degradation of 18-, 21-, and 24-nt dsRNAs (red, green, and blue respectively) in serum. The solid black lines represent double-exponential fits to the data, the fast rate constants of which are reported in panel B. (*B*) The resulting rate constants of degradation of the 18-, 21-, and 24-nt dsRNAs in serum plotted as a function of total serum protein concentration. Solid lines indicate fits with a hyperbolic binding equation. (*C*) Steady-state FRET time traces upon degradation of 18-, 21-, and 24-nt dsRNAs (red, green, and blue respectively) in cell extract. The solid black lines represent single-exponential fits to the data, the rate constant of which are reported in panel D. (*D*) The rate constants of degradation, derived from steady-state FRET (solid circles, left y-axis) and radioactive assays (open circles, right y-axis) of the 18-, 21-, and 24-nt dsRNAs (red, green, and blue respectively) in cell extract and plotted as a function of total cell extract protein concentration. Solid lines indicate fits of the FRET-derived data with a hyperbolic binding equation. (*E*). Formation of complexes D, C2 and C1 after 2 h of incubation with radiolabeled 21-nt dsRNA with no modification (lane 1), 18-nt dsRNA (lane 2), 21-nt dsRNA (lane 3) and 24-nt dsRNA (lane 4) in HeLa cytosolic extract. Lanes 5–8 represent the respective negative controls in the absence of cell extract with the dsRNAs in the same order from left to right. Normalized fractions in percent are indicated for each boxed band; * indicates an unknown complex.

Interaction with Dicer is one of the earliest and most crucial steps in RNAi, wherein larger dsRNAs are cleaved into 21-nt fragments that Dicer then loads into the RISC complex [31–34]. To test whether our 24-nt dsRNA is an authentic substrate for Dicer, we employed both FRET assays and gel based analysis. First, we collected steady-state FRET time traces for our 18-, 21-, and 24-nt dsRNAs upon addition of purified human Dicer (**Figure 4A**). While the traces for the 18- and 21-nt dsRNAs hold steady over more than 30 min incubation time, the trace of the 24-nt dsRNA significantly increases, suggestive of an interaction with Dicer (**Figure 4A**). Next, we incubated the three dsRNAs with either human Dicer or HeLa cell extract for 200 min and analyzed the degradation products on a denaturing FRET gel (**Figure 4B**). As expected, the 24-nt dsRNA is processed by Dicer to the mature 21-nt siRNA. In HeLa cell extract, the 18-nt dsRNA exhibits a slightly stronger degradation band at ∼7 nt length than either the 21- or 24-nt dsRNAs, but with that exception the lanes are quite similar. We only observed a weak 21-nt Dicer-derived cleavage band in the 24-nt dsRNA lane, suggesting that processing by Dicer under these conditions in cell extract is slow, perhaps because the incorporation into Dicer containing protein complexes is modest for our fluorophore-labeled dsRNAs (see also **Figure 5E** and corresponding discussion below). Overall, degradation is slow over the 200 min incubation and seems common to all dsRNAs in HeLa cell extract, generating the diffuse, FL-only band that co-migrates with an ∼15 nt RNA fragment and is also observed in **Figure 2G**.

### 21- and 24-nucleotide dsRNAs are protected in cell extract

Next, we employed our steady-state FRET assay to the 18-, 21-, and 24-nt dsRNAs and extracted the rate constants from the time traces that were identified in **Figure 2** as reflective of the RNA degradation kinetics (the rising phase in fetal bovine serum and the decreasing phase in HeLa extract, **Figure 5**). The results from serum (**Figure 5B**) show that the degradation rate constants progressively decrease with length of the dsRNA (18-nt dsRNA: v_max_ = 1.2 min^−1^, Serum_1/2_ = 4.5 mg/ml; 21-nt dsRNA: v_max_ = 0.47 min^−1^, Serum_1/2_ = 7.7 mg/ml; 24-nt dsRNA: v_max_ = 0.09 min^−1^, Serum_1/2_ = 1.7 mg/ml). This trend correlates with the increasing distance of the fluorophores from the ends as well as the higher thermodynamic stability of the longer duplexes (**Figure 1**). By comparison, the decay rate constants observed in cell extract appear to be an order of magnitude slower in the FRET-based assays (filled circles in **Figure 5D**, left y-axis) and even more so using 5′ ^32^P-labeled, fluorophore-labeled dsRNAs (open circles in **Figure 5D**, right y-axis), measured in a less accurate, discontinuous radioactive assay where aliquots were taken periodically and decay products analyzed by denaturing gel electrophoresis as in **Figure 2**. Steady-state FRET assays revealed that the 18-nt dsRNA is the most rapidly degraded of the three RNAs (v_max_ = 0.09 min^−1^, HeLa_1/2_ = 0.70 mg/ml). In contrast to the serum result, the 21- and 24-nt dsRNAs exhibit the same low v_max_ values, signifying that they are degraded at about the same rate in cell extract (21-nt dsRNA: v_max_ = 0.02 min^−1^, HeLa_1/2_ = 0.02 mg/ml; 24-nt dsRNA: v_max_ = 0.02 min^−1^, HeLa_1/2_ = 0.17 mg/ml). The same trend was observed using the 5′ ^32^P-radiolabeled dsRNAs (18-nt dsRNA: v_max_ = 0.011 min^−1^, HeLa_1/2_ = 0.68 mg/ml; 21-nt dsRNA: v_max_ = 0.0031 min^−1^, HeLa_1/2_ = 0.3 mg/ml; 24-nt dsRNA: v_max_ = 0.0032 min^−1^, HeLa_1/2_ = 0.29 mg/ml). These results suggest that the size dependence of the protection of dsRNA from decay in cell extract is distinct from that in serum. It should be noted that we also observed that the deoxythymidine and fluorophore modifications offered a general, modest protection to the RNAs, as may be expected (data not shown).

### Longer fluorophore labeled RNAs are more efficiently assembled into RISC-related complexes

To investigate the ability of the fluorophore labeled 18-, 21-, and 24-nt dsRNAs to form protein complexes upon incubation with HeLa cell extract we performed gel electrophoretic mobility shift assays under non-denaturing conditions to identify such complexes based on a modified RISC assembly assay (**Figure 5E**) [35]. Characteristic RISC-related RNA:protein complexes D (Retention factor, Rf: 0.2), C2 (0.4) and C3 (0.5) were observed, similar to those found previously using the unmodified 21-nt dsRNA [35]. Complex D in particular is known to contain Dicer [35,36]. We observed that the fraction of the fluorophore-labeled ^32^P-labeled RNAs incorporated into Complex D is less than that of ^32^P-labeled 21-nt dsRNA devoid of any modification, consistent with the slow processing of 24-nt dsRNA by Dicer (**Figure 4B**), whereas complexes C1 and C2 are more highly populated (**Figure 5E**). Importantly, the fraction of 18-mer dsRNA incorporated into any of these complexes is significantly less than those of either the 21-mer or 24-mer dsRNA, which may explain the comparatively lesser protection of the 18-mer relative to the 21-mer and 24-mer dsRNAs in HeLa cell extract.

## Discussion

Potent and specific induction of mRNA degradation by siRNAs is a key determinant for harnessing RNAi in reverse genetic studies and therapeutic applications. It remains unclear, however, just how stable siRNAs are inside the cell. We have previously shown the decreased nuclease sensitivity of dsRNA compared to ssRNA in human HeLa cytosolic cell extracts [26], and recent work has employed FRET fluctuation spectroscopy to demonstrate this trend inside cells [23]. We here have established real-time assays to measure degradation profiles and kinetics for small, RNAi-related RNAs in both blood serum and RNAi-active cytosolic cell extract. We show that FRET-labeled RNAs interact with relevant cellular enzymes including RNase H1 and Dicer. A DNA/RNA hybrid is strongly protected in serum, but rapidly degraded by RNase H1 in cell extract. 21- and 24-nt double-stranded RNAs are equivalently protected in HeLa cell extract, but degraded considerably faster in blood serum with their relative decay rate constants correlating with their relative lengths. Given that the 24-nt dsRNA is a substrate of human Dicer and the 21-nt dsRNA its product, these results suggest that RNAs that are involved in RNAi are specifically protected in the cellular milieu. Our observations may thus explain the relative longevity of intracellular RNAi effects induced even by chemically unmodified siRNAs [8].

As expected, dsRNAs are more stable against nucleases than the corresponding ssRNA in both HeLa cell extract and blood serum (**Figure 2**). These results are consistent with the relative stiffness of the RNA duplex, stabilizing the phosphodiester and ribose torsion angles in conformations less amenable to the in-line attack conformation needed by ribonucleases such as the RNase A-like enzymes dominant in serum [29,37]. Hybridization to a DNA further stabilizes the RNA component in blood serum, perhaps due to perturbation of the A-form helical structure or lack of 2′-OH functionality on an entire strand of the duplex. By contrast, in cell extract the DNA/RNA hybrid is rapidly degraded by RNase H1, as shown by enzyme-specific inhibition by two *in vitro* selected DNA aptamers [30] (**Figure 3**). In human cells, a second, disparate, multi-subunit enzyme, termed RNase H2, has been characterized that degrades DNA/RNA hybrids [38]. This may be the enzyme that causes the residual DNA/RNA decay upon maximal inhibition of RNase H1 (**Figure 3**).

The size range of the dsRNAs we tested for their stability in serum and cell extract was chosen to bracket the specific 21-nt size of siRNAs in human cells. dsRNAs larger than ∼21 nt are cleaved by human Dicer into siRNAs during the initial step of RNAi, as observed here for the 24-nt dsRNA, followed by incorporation into the RISC-loading complex (RLC), comprising the proteins Dicer, Ago2, and TRBP [33,34]. Since cellular delivery of 21-nt siRNAs induces RNAi, albeit not as effectively as does delivery of Dicer substrates [27], both types of dsRNAs must be interacting with the components of the RNAi machinery, in contrast to shorter dsRNAs. The similar protection of 21- and 24-nt dsRNAs in our assays relative to the corresponding 18-nt dsRNA (**Figure 5**) is consistent with these findings. In addition, the fraction of 18-mer dsRNA incorporated into RISC-related protein complexes is significantly less than those of either the 21-mer or 24-mer dsRNAs (**Figure 5E**). This result suggests that the comparatively faster degradation rate of the 18-mer versus the 21-mer and 24-mer dsRNAs may be linked to the existence of protective RNA:protein complexes in HeLa cell extract preferably for the longer dsRNAs. By contrast, the efficiency of dsRNA degradation in blood serum monotonically decreases with increasing RNA size, that is, with distance of the FRET fluorophores from the ends and overall thermodynamic stability of the duplex (**Figure 5**). These observations are in line with the known properties of RNase A, where the limiting step of enzyme action on dsRNA substrates is invasion of the duplex to access an in-line attack conformation and cleave the RNA backbone [37].

In summary, our results are consistent with established steps in the RNAi pathway where dsRNAs of ∼21 nucleotides per strand are bound by the RLC or other RNAi components and remain largely hidden from nucleolytic decay either as the base-paired siRNA duplex or as the single guide strand processed into active RISC. The real-time *in vitro* FRET assays established here open up the possibility for future high-throughput screening of the nucleolytic stability of additional chemically modified RNAi effector molecules.

## Materials and Methods

### Nucleic acid synthesis and labeling

All RNAs were synthesized by the HHMI Biopolymer/Keck Foundation Biotechnology Resource Laboratory at the Yale University School of Medicine, deprotected, TMR labeled (as necessary), and purified as previously described [39–41]. More specifically, the guide strand was labeled during synthesis with Fluorescein dT and Amino-Modifier C6 dT in the positions indicated in **Figure 1**; the amino-modifier was subsequently coupled to TMR succinimidyl ester (Invitrogen), the RNA purified away from free dye by C18-reversed phase HPLC, and its purify checked by denaturing polyacrylamide gel electrophoresis [39–41]. Sequences are derived from the firefly luciferase gene and have been shown to be effective in RNAi-mediated target cleavage [12,28]. All RNAs were purchased with a 5′ phosphate group, except for a second fluorophore-labeled 21-nt guide strand with a 5′ OH used for radiolabeling. The DNA oligonucleotides used in our studies (Sequence  =  VI-2, V-2, and S1-DNA) [30] were synthesized by Invitrogen and had the following sequences: VI-2, CGGTCGCTCCGTGTGGCTTGGGTTGGGTGTGGCAGTGAC; V-2, GCTGGTCTCTGCGGGTTGTTGCGCCGCGGCACCCTTGGCA; S1-DNA, CGTACGCGGAATACTTTGAAA.

### Steady-state FRET assays of RNA degradation

A final concentration of 50 nM FL and TMR doubly-labeled RNAs was annealed either alone (single-stranded RNA) or with 100 nM (final concentration, in excess for saturation) of an appropriate DNA or RNA complement by heating to 70°C for 2 min and cooling to room temperature over 10 min in a standard buffer of 50 mM Tris-HOAc, pH 7.4, 80 mM KCl, 20 mM NaCl, and 1 mM MgCl_2_ (chosen to mimic physiologic conditions). After the 10-min cooling period at room temperature, RNA solutions were incubated in a circulating water bath at 37°C for 5 min before the samples were added to a fluorometer cuvette, also controlled at 37°C. After the initiation of collection of donor (F_520_) and acceptor fluorescence emission signal (F_585_) as described previously [41], a baseline measurement was recorded for approximately 50–100 seconds before a fraction of either HeLa S100 cytoplasmic cell extract (a gift from Danny Reinberg, Department of Biochemistry, Rutgers University, prepared and dialyzed following published protocols [42]), fetal bovine serum (Gibco), or purified Dicer (Stratagene, 2 Units) was added and the resulting signal evolution recorded. The FRET ratio Q (F_585_/(F_585_+F_520_)) was plotted over time t and fit where appropriate with either the single- or double-exponential form of the following expression to extract the observed rate constants k_obs_ (please note that in case of a decreasing data curve the pre-exponential factor A is positive, otherwise negative):
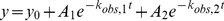
(Eq.1)


These assays were carried out over a range of 0–30% (v/v) HeLa cell extract and serum volume additions, equivalent to 0–2.5 mg/ml total HeLa protein (concentration measured by Bradford assay) and 0–11.1 mg/ml total serum protein (concentration provided by Gibco). The observed rate constants were plotted as a function of total protein content and fit with the following hyperbolic binding equation that does not assume cooperativity:
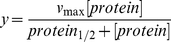
(Eq.2)


In this equation, *v_max_* is the maximal rate constant at saturation, and *protein_1/2_* is the half-titration point.

### RNase H1 inhibition assays

Fluorometer assays to monitor RNase H1 inhibition by DNA aptamers VI-2 and V-2 [30] were conducted identically to the fluorometer assays described above with the following exceptions. The HeLa cell extract used in these assays was pre-incubated with a defined concentration of the pre-annealed DNA aptamer or the negative control DNA S1 for 30 min at 37°C before mixing with the DNA/RNA hybrid in the fluorometer. In addition, the DNA/RNA hybrid was prepared in the presence of the respective aptamer or control DNA at a concentration equal to that in the HeLa cell extract. Again, the FRET Ratio Q for each trace was extracted and fit with a single-exponential expression as above, and the observed rate constants were plotted as a function of aptamer concentration and fit with the following hyperbolic binding equation below where no cooperativity was assumed:
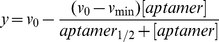
(Eq.3)


Here, *v_min_* is the minimal rate constant reached at saturation and *aptamer_1/2_* is the half-titration point.

### FRET-monitored gel electrophoretic analysis of RNA fragments

Denaturing gel electrophoresis was paired with FRET detection of the RNA to gain qualitative insight into the processing of the labeled RNAs by Dicer, cell extract, and serum. The doubly-labeled RNA strand was prepared at 500 nM final concentration in standard buffer, the complementary RNA or DNA as necessary added to 1 µM concentration and annealed as described above. After cooling, Dicer, cell extract, or serum was added as indicated and the samples were incubated at 37°C until the reactions were stopped by mixing with a final concentration of 10% (v/v) Contrad 70 at pH 9.3 (Decon Labs) as previously described [26]. For the samples of the FRET gel in **Figure 2C**, serum was added to a final total protein concentration of 11.1 mg/ml for the DNA/RNA hybrid and dsRNA, but only 0.93 mg/ml for the ssRNA. Time points for these reactions were as follows: ssRNA, 1, 5, and 10 min; DNA/RNA hybrid and dsRNA, 0.5, 1 and 2.5 h. For the samples of the FRET gel in **Figure 2G**, HeLa cell extract was added throughout to a final total protein concentration of 2.49 mg/ml and time points were taken at 5, 10, and 20 min for the ssRNA and the DNA/RNA hybrid, while time points of 5, 20, and 60 min were taken for the dsRNA. The samples for the FRET gel in **Figure 4B** were prepared by exposing each of the 18, 21, and 24-nt double-stranded RNAs to Dicer enzyme (1 unit), HeLa cell extract (2.49 mg/ml), or buffer alone for 200 min (3.3 h). The buffer for the HeLa and buffer only reactions was composed of our standard buffer supplemented with 10 mM DTT, and the Dicer reactions contained the buffer supplied and recommended by the manufacturer whose final composition was 20 mM Tris-HCl, pH 8.0, 150 mM NaCl, and 2.5 mM MgCl_2_. The T1 and OH lanes in the gels refer to reference ladders using RNase T1 and alkali (OH^−^) digestion, respectively, prepared as described [12] except that no carrier tRNA was added to the RNase T1 digest. For all reactions, 10 pmol/lane of labeled RNA was mixed with loading buffer for a final concentration of 1×TBE, 0.025% (w/v) bromophenol blue, and 40% (v/v) formamide, and the products were separated on a denaturing, 8 M urea, 20% (w/v) polyacrylamide gel. Fluorescence detection of the RNA was accomplished using a FluorImager SI fluorescence scanner with ImageQuant software (Molecular Dynamics) as previously described [43,44].

### Radioactivity-monitored gel electrophoretic analysis of RNA fragments and RISC-related RNA:protein complexes

The 21-nt non-phosphorylated, doubly-fluorophore labeled RNA was 5′-^32^P labeled with T4 polynucleotide kinase (PNK) and γ-^32^P-ATP, mixed with loading buffer to give a final concentration of 1×TBE, 0.025% (w/v) bromophenol blue, and 40% (v/v) formamide, and gel purified on a denaturing, 8 M urea, 20% (w/v) polyacrylamide gel. The radiolabeled full-length RNA was excised from the gel, diffusion eluted into 1 mM EDTA overnight, and ethanol precipitated. The dried RNA was dissolved in water.

5′-^32^P labeled RNA (50,000 cpm/lane) was mixed with 50 nM non-radiolabeled, but 5′ phosphorylated, doubly-fluorophore labeled RNA and 100 nM of the complementary 21-nt RNA strand, and annealed as described above in standard buffer. Degradation was initiated by the addition of a final total protein concentration of 2.49 mg/ml cell extract or 11.1 mg/ml serum at 37°C. For the analysis in **Figure 5D** **,** the assays were carried out over a range of 0–30% (v/v) HeLa cell extract. Time points were taken after 2, 5, 10, 20, 40, and 80 min for the cell extract sample and 5, 10, 20, 40, 80, 120, and 180 min for the serum sample. For the analysis in **Figure 5D**, time points were instead taken 2, 5, 10, 20 and 40 min after the addition of cell extract. All time points were quenched by mixing with a final concentration of 10% (v/v) Contrad 70 at pH 9.3 (Decon Labs) [26]. Loading buffer was added to a final concentration of 1×TBE, 0.025% (w/v) bromophenol blue, 0.025% (w/v) xylene cyanol, and 40% (v/v) formamide, and the degradation products separated on a denaturing, 8 M urea, 20% (w/v) polyacrylamide gel. Individual bands were identified by comparison with sequencing ladders from partial digestion with G-specific RNase T1 and alkali as described [45], quantified and normalized to the sum of all bands in a lane using a PhosphorImager Storm 840 with Image Quant software (Molecular Dynamics). Any error bars are derived from three independent determinations.

For the radioactive dsRNA degradation data in Figure 5D the normalized ratio of intact dsRNA over the sum of all bands in a lane was plotted over time *t* and fitted with a simple linear regression line to estimate the observed rate constant k_obs_:
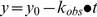
(Eq.4)


These assays were carried out over a range of 0–30% (v/v) added HeLa cell extract, equivalent to 0–2.5 mg/ml total HeLa protein (concentration measured by Bradford assay). The observed rate constants were plotted as a function of total protein content and fitted as above with hyperbolic binding equation 2.

The gel electrophoretic mobility shift assays of RISC-related RNA:protein complexes were initiated by the addition of a final total protein concentration of 2.49 mg/ml cell extract to the annealed 5′-^32^P labeled RNA in standard buffer at 4°C. After 2 h incubation the reaction was stopped by mixing with non-denaturing loading buffer to a final concentration of 1×TBE, 0.025% (w/v) bromophenol blue and 0.025% (w/v) xylene cyanol and the RNA-associated complexes were separated by non-denaturing 4% (w/v) polyacrylamide gel electrophoresis at 4^o^C, quantified and normalized to the sum of all bands in a lane using a Typhoon 9410 Variable Mode Imager with Image Quant software (GE Healthcare), essentially as described [35]. Three independent analyses gave similar results.

## Supporting Information

Figure S1 **FRET ratio time trace upon incubating doubly fluorophore labeled 21-nt dsRNA with either serum (red) or 2 ng/ml of purified RNase A (black).** The real-time FRET traces for each of these conditions reveal that both serum and the purified nuclease manifest a significant initial increase in FRET ratio.
(DOC)
