# Supplementary material for: siRNA-Like Double-Stranded RNAs Are Specifically Protected Against Degradation in Human Cell Extract
Source: PLoS One. 2011 May 27;6(5):e20359. doi: 10.1371/journal.pone.0020359 (PMC3103583; doi:10.1371/journal.pone.0020359)
Supplement: Figure S1 — FRET ratio time trace upon incubating doubly fluorophore labeled 21-nt dsRNA with either serum (red) or 2 ng/ml of purified RNase A (black). The real-time FRET traces for each of these conditions reveal that both serum and the purified nuclease manifest a significant initial increase in FRET ratio. (DOC) [file pone.0020359.s001.doc]

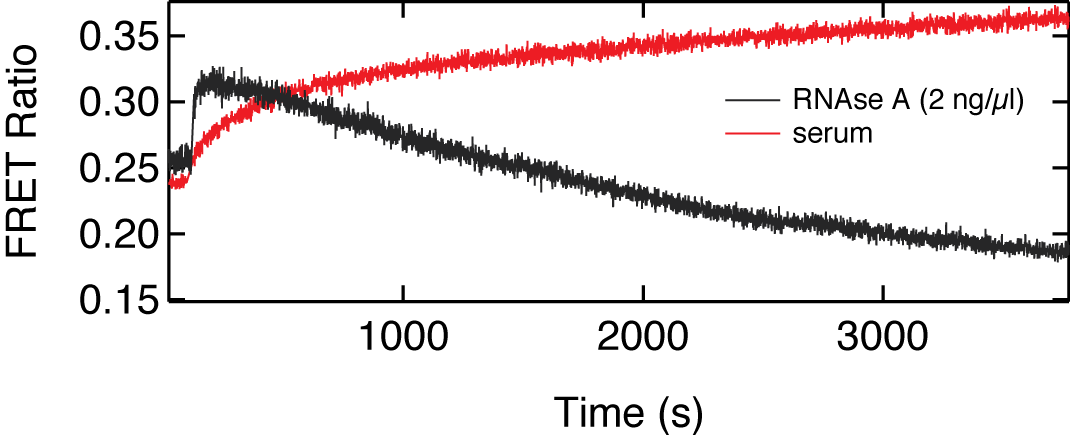


**Figure S1. FRET ratio time trace upon incubating doubly fluorophore labeled 21-nt dsRNA with either serum (red) or 2 ng/ml of purified RNase A (black).** The real-time FRET traces for each of these conditions reveal that both serum and the purified nuclease manifest a significant initial increase in FRET ratio.
